# Conventional Echocardiography and Two-Dimensional Speckle Tracking in Healthy Sevoflurane-Anesthetized Dogs Undergoing Continuous Rate Infusion of Nalbuphine

**DOI:** 10.1155/2020/9278751

**Published:** 2020-06-08

**Authors:** Marcel G. Marques, Ana Elisa G. W. Marques, Carlos E. de Siqueira, Élen A. P. de Sousa, Yan S. Ribeiro, Beatriz P. Floriano, Wagner L. Ferreira, Paulo S. P. Santos

**Affiliations:** ^1^Department of Animal Clinic, Surgery and Reproduction, School of Veterinary Medicine of Araçatuba (FVMA), São Paulo State University (UNESP), 793 Clóvis Pestana St., 16050-680 Araçatuba, SP, Brazil; ^2^Department of Veterinary Medicine of University Center of the Integrated Colleges of Ourinhos (UNIFIO), Highway BR-153, KM 338, 19909-100 Ourinhos, SP, Brazil

## Abstract

Nalbuphine is an agonist-antagonist opioid with adequate analgesic properties and few depressant effects on the respiratory system. However, there are no detailed reports available on cardiovascular effects of nalbuphine in dogs. The aim of this study was to assess the effects of a continuous rate infusion (CRI) of nalbuphine on left ventricular systolic and diastolic function of healthy sevoflurane-anesthetized dogs. Eighteen mixed-breed bitches aged 1–4 years and weighing 9.9 ± 3.8 kg were used. Dogs were randomly assigned to one of two groups: nalbuphine (G_N_, *n* = 9) and control (G_C_, *n* = 9). Anesthesia was induced and maintained with sevoflurane (2V%) followed by an intravenous (IV) bolus of nalbuphine (0.3 mg/kg) or 0.9% NaCl at equal volume and then CRI of nalbuphine (0.4 mg/kg/h) or 0.9% NaCl at an equal infusion rate. Echocardiographic and hemodynamic variables were determined at baseline and 20, 40, 60, and 80 minutes following start of CRI. No differences were found between groups for left ventricular systolic and diastolic variables obtained through conventional echocardiography and two-dimensional speckle tracking. Likewise, hemodynamic variables did not differ between groups. The E′/A′ ratio significantly increased at 20 minutes compared to baseline only in GN. Nalbuphine given at a CRI does not influence left ventricular systolic and diastolic function in healthy sevoflurane-anesthetized dogs.

## 1. Introduction

Nalbuphine is an opioid drug that acts as an agonist to *κ* receptors and antagonist to *μ* receptors, with potency and clinical analgesic effects similar to that of morphine [1]. An important advantage of nalbuphine is its minimal depressant effect on respiratory function. Nalbuphine can also be used to reverse the respiratory depression caused by full *μ* agonists, while still providing desirable analgesia [2].

Some studies have shown that mammalian myocardium has opioid receptors *μ*, *σ*, and *κ* [3–5]. One of the most important myocardium opioid receptors is the kappa. Once stimulated, they can decrease the sarcoplasmic concentrations of calcium, leading to negative inotropic effects. One laboratorial study with adult rat left ventricular myocytes has shown that an opioid substance (U-50,488H) with *κ* agonist properties can impair cardiac contractility [6]. Therefore, as the nalbuphine acts as a *κ* agonist receptor, it is believed that it can act directly on myocardial dynamic. However, no studies were found addressing the effects of a CRI of nalbuphine on left ventricular systolic and diastolic function of healthy dogs. A few studies demonstrated a decrease in mean arterial pressure (MAP) and heart rate (HR) with the use of nalbuphine in dogs [2, 7].

Among the methods currently used to assess ventricular dynamics, echocardiography stands out for being a noninvasive approach that allows real-time observation of systolic and diastolic function [8]. Also, the possibility of assessing myocardial deformity through two-dimensional speckle tracking has made it possible to further understand myocardial dynamics. This technique provides optimal imaging of myocardial deformation in their variable orientations and is highly accurate for the detection of early changes compared to conventional echocardiographic parameters [9, 10]. Other authors also demonstrated the efficacy of conventional and two-dimensional speckle tracking when assessing the effects of opioids on human and canine myocardial function [11, 12].

Therefore, the objective of this study was to assess the effects of a CRI of nalbuphine on left ventricular systolic and diastolic function of healthy dogs anesthetized with sevoflurane. The hypothesis was that nalbuphine at 0.4 mg/kg/h, when compared to a CRI of saline, would not produce clinically relevant changes on echocardiographic parameters reflecting left ventricular systolic and diastolic function of sevoflurane-anesthetized dogs.

## 2. Materials and Methods

### 2.1. Animals

Eighteen mixed-breed bitches aged 1–4 years and weighing 9.9 ± 3.8 kg were enrolled in the study. Dogs were considered healthy based on physical examination (mucous membrane color, capillary refill time, heart rate, pulmonary auscultation, and rectal temperature), cardiovascular assessment (echocardiography and electrocardiography), complete blood count, biochemical analysis (blood urea nitrogen, creatinine, alanine aminotransferase, and alkaline phosphatase), and leishmaniasis test. Changes in any of these parameters were considered as exclusion criteria for the study. The study was approved by the local Ethics Committee for Animal Usage (protocol no. 00385/2018), and treatment of the animals was appropriated.

### 2.2. Study Design

Dogs were randomly selected to participate in one of the two experimental groups, G_N_ (*n* = 9) and G_C_ (*n* = 9). G_N_ that received an IV bolus of nalbuphine (0.3 mg/kg) followed by CRI (0.4 mg/kg/h). G_C_ received a bolus and CRI of saline at equal volume and infusion rate of G_N_. The order of participation was determined by use of an online random number generator (http://www.randomization.com). The mean age of the subjects in G_N_ was 1.6 ± 0.9 years and in G_C_ was 2.4 ± 1.0 years. Mean weight in G_N_ and G_C_ was, respectively, 10.2 ± 4.1 kg and 9.6 ± 3.6 kg.

Prior to each procedure, animals were fasted for 8 hours and water was given ad libitum [13]. Preparation comprised clipping the thoracic area for echocardiographic examination, the metatarsus for invasive blood pressure monitoring, and the forearm for intravenous fluid or drug administration. Animals were kept in the exam room at 23°C for 20 minutes prior to the procedures to minimize stress and interferences with cardiac variables.

Anesthesia was then induced via facial mask with sevoflurane (Sevocris 1 mL/mL, Cristália–Produtos Químicos Farmacêuticos Ltda, Itapira, São Paulo State, Brazil) at 5V% in 100% oxygen at 5 L/min. Following loss of mandibular tonus and laryngotracheal reflex, subjects were intubated and coupled to the anesthetic circuit to receive sevoflurane at 2V% (0.85 minimum alveolar concentration for sevoflurane in dogs) in 100% oxygen at 50 mL/kg/minute. Sevoflurane concentrations were monitored by using a digital gas analyzer and adjusted to maintain anesthesia at stage III, 3^rd^ plane. The dorsal metatarsal artery and the cephalic vein were catheterized for invasive blood pressure monitoring and fluid administration, respectively. Catheters were coupled to a PRN adaptor and flushed with heparinized saline.

With animals fully instrumentalized, anesthesia was stabilized for 15 minutes prior to data collection and animals were allowed to breathe spontaneously throughout the study. Subsequently, baseline variables were recorded, followed by an intravenous (IV) bolus of nalbuphine (Nubain 10 mg/mL, Cristália–Produtos Químicos Farmacêuticos Ltd, Itapira, São Paulo State, Brazil) (0.3 mg/kg, G_N_) or saline at equal volume (G_C_) and CRI of nalbuphine (0.4 mg/kg/h) or saline at equal infusion rate. Infusions were administered by an infusion pump (Syringe pump 680–Samtronic, São Paulo, São Paulo State, Brazil) in both groups.

Data collection happened at baseline and 20, 40, 60, and 80 minutes following start of CRI. Hemodynamic variables comprised HR, obtained directly from a multiparameter monitor (Dixtal—mod. DX-2020—Manaus, Amazonas State, Brazil), and invasive arterial blood pressure, systolic arterial pressure, mean arterial pressure, and diastolic arterial pressure (SAP, MAP and DAP), recorded from the same monitor with the transducer zeroed at heart level. The multiparameter monitor was also used to record SpO_2_ and a digital thermometer was used to measure rectal temperature.

### 2.3. Conventional Echocardiography

Conventional echocardiography (MyLab 30 Gold VET, Genoa, Italy) was performed using a multifrequency transducer (1–4 MHz and 7.5–10 MHz) with simultaneous electrocardiographic monitoring. One examiner (MGM) who was unaware of the treatment performed the recordings according to preestablished standards [8] at the left parasternal window for the four-chamber apical view. End-diastolic and end-systolic volumes and ejection fraction (EF) were obtained following the planimetric Simpson method [8, 14]. Volumes were indexed using the body surface area (BSA) to obtain the end-diastolic volume index (EDVI) and end-systolic volume index (ESVI) [15]. By placing the Doppler cursor on the extremity of the mitral valve, peak velocity of early left ventricular filling (E wave) and atrial contraction (A wave) were measured and the E : A ratio was calculated. Using a five-chamber apical view with the Doppler cursor on the midpoint between left ventricular outflow and transmitral flow, the isovolumetric relaxation time (IVRT) was recorded. Finally, the velocity-time integral (VTI) was obtained with the cursor placed distally to the aortic valve.

Tissue Doppler assessment was performed on the four-chamber apical view with the sample volume placed on the lateral border of the mitral annulus. Two negative peak velocities were then obtained: peak early left ventricular filling (E′ wave) and atrial contraction (A′ wave), and the E′ : A′ ratio was calculated. The S′ wave was determined as the positive peak during ventricular systole.

At the right parasternal window, the aortic valve area (AVA) was measured at the two-dimensional view of the left ventricle in its short axis and the transducer inclined to allow visualization of the aortic plane. The AVA was measured during the early diastole, after the closure of the aortic cups.

Recorded variables were further used to calculate Doppler ejection index (DEI), Doppler cardiac index (DCI), and peripheral vascular resistance index (PVRI), as follows:

DEI (mL/beat/m^2^) = (VTI [cm] × AVA [cm^2^])/BSA, where BSA = weight (g)^0.67^/1000; DCI (L/minute/m^2^) = (DEI [mL/m^2^] × HR [beats/minute])/1000; and PVRI (dyne x s/cm^5^/m^2^) = (MAP [mmHg]/DCI [L/minute/m^2^]) × 80.

### 2.4. Two-Dimensional Speckle Tracking Echocardiography

**Figure 1:**
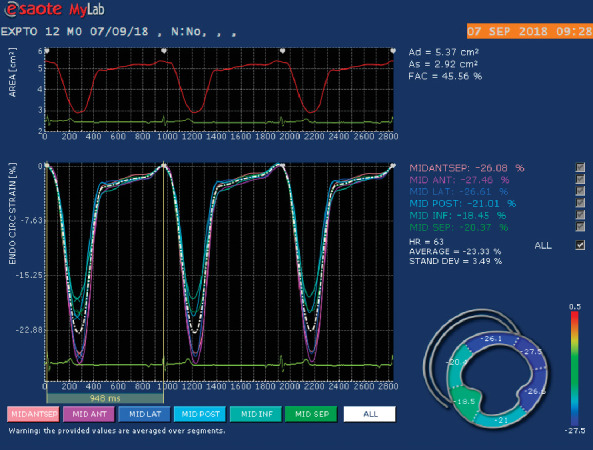
Representative 2D echocardiographic image obtained from a healthy dog providing a right parasternal short-axis view of the left ventricle at the level of the papillary muscles in which there is a region of interest drawn by the software that has been adjusted manually. The myocardium has been automatically divided into 6 segments for strain analysis: anterior, anterolateral, inferolateral, inferior, inferoseptal, and anteroseptal. The bottom panel provides color-coded circumferential strain curves for each of the 6 segments and a global strain curve (dotted white line), with strain value on the *y*-axis and time in seconds on the *x*-axis. Strain values for each segment are indicated and represent the corresponding percentage.

**Figure 2:**
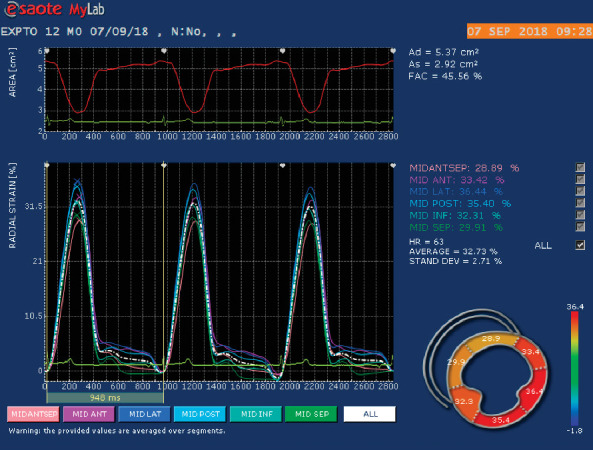
Representative 2D echocardiographic image obtained from a healthy dog providing a right parasternal short-axis view of the left ventricle at the level of the papillary muscles in which there is a circular region of interest drawn by the software that has been adjusted manually (bottom left). The myocardium has been automatically divided into 6 segments for strain analysis: anterior, anterolateral, inferolateral, inferior, inferoseptal, and anteroseptal. The bottom panel provides color-coded radial strain curves for each of the 6 segments and a global strain curve (dotted white line), with strain value on the *y*-axis and time in seconds on the *x*-axis. Strain values for each segment are indicated and represent the corresponding percentage.

The same ultrasound machine was used to assess two-dimensional speckle tracking with a multifrequency transducer of 1–4 MHz and 7.5–10 MHz and simultaneous electrocardiography. One examiner (MGM) who was unaware of the treatment was responsible for obtaining and analyzing the images. Only optimal images obtained between 50 and 110 Hz for at least three consecutive cycles were used. Images were stored digitally for further offline analysis using specific software (MylabTM Desk algoritmo X-strainTM, Genoa, Italy). This echocardiographic machine uses a speckle tracking software (feature tracking) that is based on a monodimensional technology that samples smaller speckles. This software calculates epicardial and endocardial strain and strain rate variables, not directly global strain and strain rate variable. Circumferential and radial strain and systolic strain rate were obtained from the left ventricular short axis at the level of the papillary muscles. Three points were manually inserted, first on the level of the posteromedial papillary muscle and second at lateral wall. The software then traced the area of interest comprising the endocardial and epicardial margins of the left ventricle. If a given point had been misplaced, the examiner performed manual corrections to ensure all margins of interest were included. The algorithm of the software then automatically segmented the left ventricle and performed myocardial tracing along six segments: anterior, anterolateral, inferolateral, inferior, inferoseptal, and anteroseptal. Six strain and systolic strain rate profiles were obtained, which corresponded to the mean values of each myocardial segment. The mean values of all six segments were used to calculate the global strain and systolic strain rate, as follows: global circumferential strain (GCS), global radial strain (GRS), global circumferential systolic strain rate (GCSSR), and global radial systolic strain rate (GRSSR) (Figures 1 and 2).

**Figure 3:**
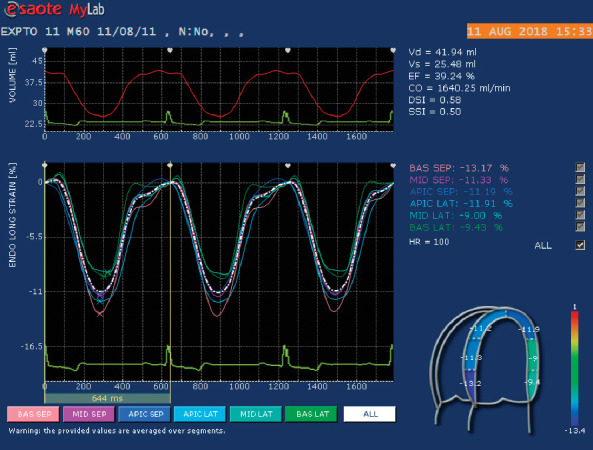
Representative 2D echocardiographic image obtained from a healthy dog providing a left apical 4-chamber view of the left ventricle in which a region of interest with delineation of endocardial border has been adjusted manually. The myocardium has been automatically divided into 6 segments for strain analysis: basal septal, middle septal, apical septal, apical lateral, middle lateral, and basal lateral. The bottom panel provides color-coded longitudinal strain curves for each of the 6 segments, with strain value on the *y*-axis and time in seconds on the *x*-axis. Strain values for each segment are indicated and represent the corresponding percentage.

Longitudinal strain and systolic strain rate were obtained from the four-chamber apical view with two points manually placed on the left ventricle, one at each side of the mitral annulus, and the third point on the apical region of the endocardial margin. The software then traced the region of interest and the examiner adjusted the points as needed. The left ventricle was divided into six segments: basal septal, middle septal, apical septal, apical lateral, middle lateral, and basal lateral. The algorithm of the software traced the left ventricle and provided values for strain and systolic strain rate. The mean values of all six segments were used to calculate the global strain and systolic strain rate, as follows: global longitudinal strain (GLS) and global longitudinal systolic strain rate (GLSSR) (Figure 3).

The intraobserver repeatability test for speckle tracking variables was determined by use of 9 healthy dogs on 3 days over a 1-week period. Each variable (strain and strain rate) was measured during 3 consecutive cardiac cycles using the same loop. The mean value was used to determine the coefficient of variation (standard deviation/mean).

### 2.5. Statistical Analysis

Variables were first tested for normal distribution using the Shapiro–Wilk test. Group comparisons were performed using two-way analysis of variance (ANOVA), and time comparisons were performed using ANOVA for repeated measures, followed by Dunnett's post hoc test for multiple comparisons. Differences were considered significant when *P* < 0.05. All analyses were performed using commercial software (GraphPad Prism, Inc. version 8.2.0 2019). Intraobserver repeatability tests were performed for two-dimensional speckle tracking variables as variation coefficient = (standard deviation/mean) × 100.

## 3. Results

Variables reflecting left ventricular volumes and systolic function, such as EDVI, ESVI, EF, DEI, DCI, and S′ wave did not differ among time points compared to baseline or between groups (Table 1). Speckle tracking variables reflecting systolic function, such as GCS, GCSSR, GRS, GRSSR, GLS, and GLSSR, were not different between groups or time points (Table 2). With regard to diastolic function, only the E′ : A′ ratio showed a significant increase at 20 minutes compared to baseline in G_N_. Nevertheless, there were no differences between groups (Table 3).

Hemodynamic variables HR, MAP, SAP, DAP, and PVRI did not differ between groups or time points (Table 4) and SpO_2_ remained at 100% throughout the experiment in both groups. Also, forced air warming mattress was used to maintain the rectal temperature at the values between 36.5 and 38.5°C in both groups.

Intraobserver repeatability testing showed a variation coefficient of 1.58–18.95% for two-dimensional speckle tracking variables.

## 4. Discussion

According to the current literature, this study is the first to assess left ventricular systolic and diastolic function of dogs undergoing a CRI of nalbuphine. The results showed that nalbuphine did not produce clinically relevant changes on echocardiographic parameters of healthy sevoflurane-anesthetized dogs. The combined use of conventional echocardiography and speckle tracking allowed a detailed analysis of the effects of nalbuphine on myocardial dynamics. Despite being considered safe from a cardiovascular point of view, opioids when combined with other drugs can cause significant changes in cardiovascular function, especially when associated with volatile anesthetics [16]. However, this study demonstrated that in addition to the known advantages of nalbuphine compared to other opioid drugs, like its minimal depressant effect on respiratory function, there is no influence of the drug on left ventricular function of healthy dogs, which renders nalbuphine a safe choice from a cardiovascular standpoint.

Echocardiography has been used previously in animals and humans to assess the effects of various drugs on cardiac function [17–20]. Several echocardiographic methods can be used for hemodynamic monitoring [17, 21] and its main advantage is the safety of its noninvasive approach. In addition, it allows real-time observation of many parameters of the cardiac cycle, thus improving comprehension of left ventricular systolic and diastolic function.

Myocardial deformation occurs in different orientations. The combined action of longitudinal, radial, and circumferential myocardial deformation enables the heart to function properly. Individual assessment of each deformation orientation provides a complete understanding of myocardial dynamics [22]. Therefore, this study used two-dimensional speckle tracking to assess deformation (strain) and deformation rate (strain rate) of the three main orientations of myocardial deformations. Furthermore, the technique is highly sensitive in detecting early systolic dysfunction compared to conventional methods used in echocardiography [10]. One study showed that by assessing strain and strain rate of dogs undergoing general anesthesia, left ventricular systolic function can be accurately investigated. Therefore, speckle tracking can replace invasive monitoring methods in dogs [23]. Two-dimensional speckle tracking has also proved useful to assess the effects of opioids on cardiac function in both animals and humans [11, 12].

Variables reflecting left ventricular systolic and diastolic function obtained through conventional echocardiography and speckle tracking did not change during infusion of nalbuphine, compared to G_C_. These findings demonstrate clearly that nalbuphine at 0.4 mg/kg/h does not influence systolic function of healthy dogs anesthetized with sevoflurane. Previous studies showed that mammalian myocardium can be directly influenced by opioids, given the presence of opioid receptors *μ*, *σ* and *κ* [3–5]. Kappa receptors seem to be the main regulators of cardiac function. By stimulating *κ* receptors, the sarcoplasmic concentrations of calcium decrease, which impairs cardiac contractility [6]. However, although nalbuphine acts as a *κ* receptor agonist, the stability found in this study can be ascribed to the infusion rate. Other authors showed that higher infusion rates can reduce contractility and influence systolic function directly [24]. One study demonstrated that an IV bolus of 0.5 mg/kg was capable of producing minimal reductions in a few hemodynamic variables [7], although systolic function was not assessed through echocardiographic examination. Therefore, it is not possible to assume that the effects of nalbuphine on left ventricular systolic function are dose-dependent.

Both EF and S′ wave were close to the lower acceptable limit for the canine species [15] in G_N_ and G_C_. However, there was no different between groups. This could be an effect of sevoflurane anesthesia, not nalbuphine infusion. Volatile anesthetic agents are known to inhibit sodium and calcium exchange in myocardial, resulting in a negative inotropic effect [25]. Similar findings were reported by other authors, thus demonstrating that volatile anesthesia decreases some of the echocardiographic variables of healthy dogs and should be used with caution in patients with systolic dysfunction [17, 18].

While speckle tracking is a validated technique [26] that has been widely used in dogs [9], the current literature shows a significant discrepancy regarding the values of strain and systolic strain rate obtained using software from different manufacturers [27]. The discrepancy hinders the establishment of reference values for the canine species, thus compromising interpretation of the results obtained in this study. However, since no differences were found between treatments, it is possible to infer that nalbuphine at 0.4 mg/kg/h does not influence global dynamics of longitudinal, radial, and circumferential myocardial deformations during sevoflurane anesthesia. Another study using the same technique also demonstrated that another *k* agonist opioid does not produce relevant changes on left ventricular function, thereby preserving global indices measured by two-dimensional speckle tracking [11, 12].

The results of HR and systolic function explain the stability seen on DEI and DCI in both treatments. As a consequence, SAP, DAP, and MAP remained stable throughout the infusions in G_N_ and T_C_. Therefore, as previously discussed, nalbuphine provides hemodynamic stability when given at a CRI for 80 minutes. These findings corroborate other studies showing that even higher doses of nalbuphine (0.5 mg/kg) did not produce important changes on HR and MAP in dogs [7]. In addition, one study in humans demonstrated that patients from the intensive care unit who received nalbuphine showed desirable analgesia with hemodynamic stability compared to those receiving sufentanil [28]. Other authors, however, demonstrate significant reductions of HR and arterial pressure using CRI of nalbuphine during inhalation anesthesia in rats with sepsis [29].

Transmitral flow and mitral annular motion were used to assess the effects of nalbuphine on left ventricular diastolic function. The study of diastolic function is crucial, since a dysfunction in ventricular relaxation can influence or precede systolic dysfunction [30]. None of the echocardiographic parameters related to diastolic function differed between groups, demonstrating that nalbuphine does not interfere with left ventricular diastolic function.

Doppler transmitral flow analysis showed slightly lower values of A wave velocity compared to reference limits in both groups along all time points [31]. This result is likely explained by sevoflurane anesthesia, since its negative inotropic effect could have decreased atrial contractility, thereby decreasing the A wave velocity. For that reason, the E : A ratio was high (E : A > 2) in practically all measurements in both groups. However, given that atrial contractility itself was not assessed in this study, these assumptions could not be confirmed. In addition, it is known that volatile anesthetics can promote significant changes in left ventricular relaxation and filling. Sarkar et al. showed that volatile anesthetics improved left ventricular relaxation due to possible reduction in afterload [32]. These authors conclude that the positive effects of volatile anesthetics on left ventricular relaxation can have an interesting effect on the management of patients with diastolic dysfunction. Nevertheless, these changes were not clinically relevant in this study, since the E : A ratio remained within acceptable limits for the species (0.92–2.72) [31]. In addition, the use of pulsed tissue Doppler showed that E′ wave velocity remained stable in nalbuphine-treated animals. This demonstrates, as described elsewhere [31], that the active process of myocardial motion during diastole remained stable even under the effects of nalbuphine and sevoflurane. Other parameters from spectral and tissue Doppler analysis also remained within acceptable ranges for dogs [31].

A few limitations are worth mentioning in this study. While 18 subjects are statistically sufficient to detect differences among treatments and time points, a higher number of subjects could elicit significant changes that could not be evidenced in this study. Furthermore, speckle tracking, though an advantageous technique, is extremely dependent on two-dimensional high-resolution imaging, which was not always possible, thus hindering the process of tracking some myocardial points and increasing the standard deviation of the results. Finally, the use of sevoflurane limits assessment of the effects of nalbuphine alone on left ventricular systolic and diastolic function.

## 5. Conclusions

Nalbuphine given at a continuous rate infusion of 0.4 mg/kg/h did not influence conventional and two-dimensional speckle tracking echocardiographic parameters of left ventricular systolic and diastolic function in healthy sevoflurane-anesthetized dogs along 80 minutes of observation [33, 34].

## Figures and Tables

**Table 1:** Mean ± standard deviation of echocardiographic variables reflecting left ventricular volumes and systolic function in 18 bitches anesthetized with sevoflurane and undergoing continuous rate infusion of nalbuphine (G_N_) or saline (G_C_) over 80 minutes of observation.

Variable	Group	Time (minutes)				
		Baseline	20	40	60	80
EDVI (mL/m^2^)	G_N_	34.9 ± 9.6	34.1 ± 10.3	34.7 ± 8.8	33.3 ± 7.6	35.2 ± 9.7
	G_C_	32.5 ± 6.0	33.3 ± 4.8	32.7 ± 4.7	32.3 ± 6.6	33.3 ± 5.1

ESVI (mL/m^2^)	G_N_	16.7 ± 5.0	15.7 ± 5.2	15.5 ± 3.5	15.1 ± 3.4	15.8 ± 4.4
	G_C_	15.5 ± 3.2	14.3 ± 2.3	14.8 ± 3.5	14.7 ± 3.5	15.6 ± 2.9

EF (%)	G_N_	51 ± 9	53 ± 8	55 ± 9	54 ± 8	54 ± 7
	G_C_	52 ± 7	57 ± 6	55 ± 9	55 ± 6	53 ± 7

S′ wave (m/s)	G_N_	0.07 ± 0.02	0.08 ± 0.02	0.09 ± 0.03	0.09 ± 0.03	0.08 ± 0.03
	G_C_	0.08 ± 0.03	0.08 ± 0.02	0.08 ± 0.02	0.08 ± 0.02	0.08 ± 0.02

DEI (mL/beat/m^2^)	G_N_	37.7 ± 8.1	42.9 ± 9	43.1 ± 8.5	44.8 ± 10.1	43.3 ± 9.5
	G_C_	32.9 ± 10.0	34.1 ± 11.5	33.7 ± 10.6	32.5 ± 9.2	34.1 ± 11.3

DCI (L/minute/m^2^)	G_N_	4.0 ± 1.3	3.9 ± 1.7	4.1 ± 1.46	4.4 ± 2.1	4.1 ± 1.7
	G_C_	3.5 ± 1.2	3.5 ± 1.3	3.5 ± 1.2	3.2 ± 1.0	3.5 ± 1.2

Variables do not differ according to ANOVA (*P* > 0.05). EDVI, end-diastolic volume index; ESVI, end-systolic volume index; EF, ejection fraction; S′ wave, peak systolic mitral annular velocity derived from tissue Doppler; DEI, Doppler ejection index; DCI, Doppler cardiac index.

**Table 2:** Mean ± standard deviation of echocardiographic variables reflecting left ventricular systolic function obtained through two-dimensional speckle tracking in 18 bitches anesthetized with sevoflurane and undergoing continuous rate infusion of nalbuphine (GN) or saline (GC) over 80 minutes of observation.

Variable	Group	Time (minutes)				
		Baseline	20	40	60	80
GCS (%)	G_N_	−19.8 ± 6.8	−21.2 ± 3.9	−22.4 ± 5.9	−20.2 ± 5.3	−21.3 ± 4.4
	G_C_	−19.5 ± 3.7	−21.1 ± 5.3	−21.4 ± 4.3	−20.7 ± 4.3	−20.8 ± 4.0

GCSSR (s^−1^)	G_N_	−1.0 ± 0.7	−1.8 ± 0.5	−1.9 ± 0.7	−1.6 ± 0.6	−1.8 ± 0.6
	G_C_	−1.7 ± 0.4	−1.7 ± 0.5	−1.8 ± 0.4	−1.7 ± 0.4	−1.7 ± 0.3

GRS (%)	G_N_	23.9 ± 6.3	24.1 ± 6.5	27.8 ± 5.8	24.7 ± 6.8	27.0 ± 7.5
	G_C_	22.5 ± 6.6	30.1 ± 6.2	28.7 ± 7.1	30.6 ± 7.0	26.2 ± 6.1

GRSSR (s^−1^)	G_N_	1.4 ± 0.4	1.4 ± 0.2	1.8 ± 0.5	1.6 ± 0.4	1.6 ± 0.5
	G_C_	1.6 ± 0.3	2.0 ± 0.5	1.8 ± 0.5	1.9 ± 0.4	1.6 ± 0.4

GLS (%)	G_N_	−12.2 ± 2.1	−13.5 ± 1.8	−13.7 ± 2.6	−13.3 ± 2.7	−13.4 ± 2.1
	G_C_	−14.4 ± 3.2	−14.0 ± 2.3	−15.6 ± 1.8	−15.1 ± 2.0	−14.8 ± 2.2

GLSSR (s^−1^)	G_N_	−1.0 ± 0.2	−1.1 ± 0.2	−1.1 ± 0.2	−1.0 ± 0.2	−1.1 ± 0.2
	G_C_	−1.3 ± 0.3	−1.1 ± 0.1	−1.3 ± 0.2	−1.1 ± 0.1	−1.2 ± 0.2

Variables do not differ according to ANOVA (*P* > 0.05). GCS, global circumferential strain; GCSSR, global circumferential systolic strain rate; GLS, global longitudinal strain; GLSSR, global longitudinal systolic strain rate; GRS, global radial strain; GRSSR, global radial systolic strain rate.

**Table 3:** Mean ± standard deviation of echocardiographic variables reflecting left ventricular diastolic function in 18 bitches anesthetized with sevoflurane and undergoing continuous rate infusion of nalbuphine (GN) or saline (GC) over 80 minutes of observation.

Variable	Group	Time (minutes)				
		Baseline	20	40	60	80
E wave (m/s)	G_N_	0.64 ± 0.20	0.68 ± 0.18	0.71 ± 0.20	0.70 ± 0.19	0.68 ± 0.22
	G_C_	0.63 ± 0.09	0.63 ± 0.09	0.63 ± 0.10	0.63 ± 0.11	0.59 ± 0.10

A wave (m/s)	G_N_	0.30 ± 0.09	0.27 ± 0.09	0.34 ± 0.11	0.30 ± 0.13	0.33 ± 0.12
	G_C_	0.30 ± 0.09	0.27 ± 0.10	0.32 ± 0.08	0.28 ± 0.05	0.33 ± 0.08

E : A ratio	G_N_	2.32 ± 1.06	2.75 ± 1.15	2.28 ± 0.96	2.68 ± 1.39	2.30 ± 1.16
	G_C_	2.23 ± 0.59	2.59 ± 1.00	2.03 ± 0.42	2.34 ± 0.61	1.87 ± 0.46

IVRT (ms)	G_N_	55 ± 9	53 ± 9	60 ± 18	57 ± 13	63 ± 16
	G_C_	55 ± 6	57 ± 10	60 ± 9	59 ± 7	63 ± 12

E′ wave (m/s)	G_N_	0.11 ± 0.03	0.12 ± 0.02	0.12 ± 0.05	0.12 ± 0.03	0.13 ± 0.04
	G_C_	0.12 ± 0.03	0.12 ± 0.03	0.12 ± 0.03	0.11 ± 0.02	0.11 ± 0.03

A′ wave (m/s)	G_N_	0.06 ± 0.02	0.05 ± 0.01	0.06 ± 0.01	0.06 ± 0.02	0.06 ± 0.01
	G_C_	0.06 ± 0.02	0.06 ± 0.02	0.07 ± 0.02	0.06 ± 0.02	0.06 ± 0.02

E′ : A′ ratio	G_N_	1.94 ± 1.00	2.52 ± 1.06^*∗*^	2.14 ± 0.92	2.23 ± 1.00	2.31 ± 0.98
	G_C_	2.17 ± 0.93	2.09 ± 0.85	1.77 ± 0.52	1.94 ± 0.55	1.85 ± 0.51

^*∗*^Significantly different compared to baseline according to Dunnett's test (*P* < 0.05). E wave, peak velocity of early left ventricular filling; A wave, peak velocity of atrial contraction; IVRT, isovolumic relaxation time; E′ wave, peak early left ventricular filling derived from tissue Doppler; A′ wave, peak velocity of atrial contraction derived from tissue Doppler.

**Table 4:** Mean ± standard deviation of hemodynamic variables in 18 bitches anesthetized with sevoflurane and undergoing continuous rate infusion of nalbuphine (G_N_) or saline (G_C_) over 80 minutes of observation.

Variable	Group	Time (minutes)				
		Baseline	20	40	60	80
HR (beats/minute)	G_N_	103 ± 18	89 ± 20	94 ± 18	95 ± 23	93 ± 21
	G_C_	108 ± 17	104 ± 23	103 ± 13	99 ± 13	101 ± 10

SAP (mmHg)	G_N_	105 ± 19	99 ± 24	105 ± 20	107 ± 23	105 ± 27
	G_C_	93 ± 33	101 ± 16	100 ± 10	106 ± 12	106 ± 18

DAP (mmHg)	G_N_	57 ± 11	55 ± 12	57 ± 11	58 ± 12	58 ± 11
	G_C_	59 ± 10	57 ± 12	55 ± 6	58 ± 6	61 ± 8

MAP (mmHg)	G_N_	71 ± 12	68 ± 15	72 ± 14	72 ± 15	72 ± 13
	G_C_	74 ± 11	71 ± 13	70 ± 6	73 ± 7	76 ± 10

PVRI (dyne × s/cm^5^/m^2^)	G_N_	1575 ± 589	1540 ± 496	1525 ± 513	1446 ± 444	1597 ± 605
	G_C_	1877 ± 806	1870 ± 938	1832 ± 777	2005 ± 787	2009 ± 865

Variables do not differ according to ANOVA (*P* > 0.05). HR, heart rate; SAP, systolic arterial pressure; DAP, diastolic arterial pressure; MAP, mean arterial pressure; PVRI, peripheral vascular resistance index.

## Data Availability

The raw data used to support the findings of this study are available from the author upon request.
